# From social support to subjective wellbeing: the sequential roles of enjoyment-based exercise motivation and flow experience among physically active women

**DOI:** 10.3389/fpsyg.2026.1880314

**Published:** 2026-08-04

**Authors:** Jian Li, Weiye Kang, Shuai Li, Chao Li, Chunyang Ren, Huiling Liao

**Affiliations:** 1 Department of Physical Education, Qufu Normal University, Qufu, China; 2 Department of Physical Education, Anhui University of Finance & Economics, Bengbu, China; 3 Tangshan Preschool Teachers College, Tangshan, China; 4 Department of Physical Education, Jeonbuk National University, Jeonju-si, Republic of Korea

**Keywords:** enjoyment-based exercise motivation, flow experience, mediating role, perceived social support, subjective wellbeing

## Abstract

**Background:**

In health promotion, perceived social support has been widely regarded as an important external resource related to individual wellbeing. Women face multiple pressures in daily life, and for them, regular exercise serves not only as physical activity but also as a key factor in improving mental health and happiness. How perceived social support is associated with wellbeing through specific psychological mechanisms remains under-explored. This study examines the mediating roles of Enjoyment-Based Motivation and Flow Experience in the relationship between perceived social support and subjective wellbeing among women who engage in regular exercise.

**Methods:**

A cross-sectional survey was conducted with 361 women who engage in regular exercise. Data analysis utilized SPSS and AMOS structural equation modeling. Participants completed self-report measures assessing perceived social support, enjoyment-based exercise motivation, flow experience, and subjective wellbeing.

**Results:**

Analysis revealed that perceived social support was positively associated with enjoyment-based motivation, flow experience, and subjective wellbeing. Enjoyment-based exercise motivation was positively associated with flow experience and subjective wellbeing, while flow experience was also positively associated with subjective wellbeing. Beyond direct paths, mediation analysis identified three mediating pathways: (a) an independent mediation of Enjoyment-Based Motivation, (b) an independent mediation of Flow Experience, and (c) a chained mediation involving both.

**Discussion:**

By incorporating Enjoyment-Based Motivation and Flow Experience as mediating variables, this study provides a more detailed examination of the potential psychological pathways linking perceived social support and subjective wellbeing. These findings may have practical implications for supporting life satisfaction and wellbeing among women who engage in regular exercise.

## 1 Introduction

The pursuit of happiness is an enduring goal for every individual. The *2022 World Happiness Report* released by the United Nations indicates that the happiness index of Chinese residents ranks only 72nd in the world (Helliwell et al., 2022). Despite this progress, China still does not rank among the happiest nations. Previous studies have identified numerous factors associated with wellbeing. For instance, previous studies have suggested that physical activity may contribute to positive psychological states and subjective wellbeing (Mansfield et al., 2020; Mahindru et al., 2023). From the perspective of positive psychology, physical exercise fosters a sound physical and mental state, cognitive and personality development (Biddle et al., 2019; Piepiora et al., 2021), and positive psychological traits and subjective wellbeing.

An increasing number of people are participating in physical activities. Regular physical activity has been reported to be associated with a lower incidence of cardiovascular and muscular diseases (Sigal et al., 2018), fewer symptoms of anxiety and depression (Schuch et al., 2016; Stanton et al., 2020), and better quality of life (World Health Organization, 2024). The WHO is committed to increasing the prevalence of physical activity, with the global goal of reducing physical inactivity by 15% by 2030 relative to the 2010 baseline (World Health Organization, 2022). This aims to help countries improve the physical activity levels of their populations and reduce disease prevalence.

Women face pressures from family, work, and society (Xiao et al., 2025). They are more susceptible to developing mental or physical health issues due to life stressors or potential traumatic events (Yamada et al., 2018; Vigna et al., 2019). Their likelihood of developing depression is approximately twice that of men (Andersson et al., 2006). According to self-determination theory, social support may function as a vital external resource and may be related to women’s internal psychological resources (e.g., emotion regulation and stress resilience) by fulfilling their core psychological needs for autonomy, competence, and relatedness (Ryan and Deci, 2017). Therefore, leveraging the protective role of social support for individual wellbeing is essential. High levels of social support buffer the negative impact of stress on mental health, which makes it a key protective factor against psychological disorders such as anxiety and depression (Zhang et al., 2021; Duraku et al., 2023).

In physical activity, enjoyment-based exercise motivation and flow experience are two important psychological factors associated with an individual’s health. They play a crucial role in shaping the psychological experiences of individuals during sports participation (Wu et al., 2021). Existing research has gradually revealed the roles of enjoyment-based exercise motivation and flow experience in relation to subjective wellbeing. However, studies on how perceived social support, enjoyment-based exercise motivation, and flow experience are collectively associated with subjective wellbeing remain insufficient.

For many years, subjective wellbeing and life satisfaction have been the research focus for researchers (Iwon et al., 2021). Subjective wellbeing is studied in terms of its emotional components (positive and negative affect) and cognitive components (life satisfaction) (Xu et al., 2022). It represents the combined outcome of situational variables and self-system variables (Huebner and McCullough, 2000; Diener et al., 1999). Subjective wellbeing is correlated with many factors. Individuals with high levels of subjective wellbeing are associated with stronger immune systems and lower cardiovascular mortality rates (Alves et al., 2008). Given the numerous positive outcomes associated with subjective wellbeing, understanding factors associated with subjective wellbeing is of great importance.

Perceived social support is one of the actively researched areas in recent years. Social support is defined as the exchange of resources between individuals based on their social networks. As a subjective dimension of social support, perceived social support is defined as an individual’s belief that they can rely on a social network for assistance when needed (Klainin-Yobas et al., 2015). As one of the factors most consistently associated with subjective wellbeing (Diener and Seligman, 2002), empirical research on the relationship between perceived social support and wellbeing has been conducted from various perspectives based on social cognitive theory. Perceived social support can comprehensively assess the likelihood of social networks providing support (Demaray and Malecki, 2002). It has shown stronger associations with mental health and wellbeing (Beeble et al., 2009; Yu et al., 2020) than objectively existing social support (Robbins et al., 2004).

While the direct correlation mentioned above has been validated (Zhang and Sun, 2024; Zuo et al., 2024; Hidalgo-Fuentes et al., 2024), there remain two critical gaps in existing research. First, the exploration of the intrinsic mechanisms between the two is insufficient, and the role of mediating variables has not been clearly defined. Second, there is a scarcity of research targeting specific groups, such as women who engage in regular exercise. This demographic faces multiple pressures from family, work, and other areas (Xiao et al., 2025), which makes them more vulnerable in terms of mental health. Meanwhile, regular exercise, as a positive health behavior, may interact with perceived social support and subjective wellbeing in unique ways.

Motivation is an internal driving force prompting individuals to engage in specific activities (Kelso et al., 2020). Based on self-determination theory, exercise motivation consists of extrinsic motivation and intrinsic motivation (González et al., 2019; She et al., 2023). Intrinsic motivation arises when individuals participate in physical activity out of genuine interest or enjoyment, rather than external incentives or pressures. The stronger the intrinsic motivation, the greater the individual’s interest and sense of achievement in exercise (Peng et al., 2025). Self-determination theory suggests that individuals’ motivations are enhanced by satisfying their three psychological needs (i.e., autonomy, competence, and relatedness) (Ryan and Deci, 2000; Rodrigues et al., 2018). Although exercise motivation is an internal psychological process, self-determination theory also emphasizes that social-contextual factors can support or hinder the satisfaction of these needs (Vansteenkiste et al., 2020). In this sense, perceived social support can be understood as an external psychological resource that may help satisfy women’s needs for autonomy, competence, and relatedness, and may therefore be related to the development of enjoyment-based exercise motivation.

Within self-determination theory, exercise motivation is not a homogeneous construct. Different motivational dimensions may have different psychological implications (Ryan and Deci, 2000). Compared with externally oriented motives such as appearance, health, or social motives, enjoyment-based exercise motivation reflects intrinsic engagement in exercise for the pleasure, interest, and satisfaction derived from the activity itself. This dimension is theoretically relevant to flow experience because flow is more likely to occur when individuals are absorbed in activities that are inherently enjoyable (Markland and Ingledew, 1997; Ryan et al., 1997; Kowal and Fortier, 1999). Therefore, the present study focuses specifically on enjoyment-based exercise motivation as the motivational pathway linking perceived social support, flow experience, and subjective wellbeing.

Flow experience is an important indicator for measuring positive emotions, emphasizing full engagement to achieve a transcendent psychological state. This state is self-driven. It occurs when an individual is completely absorbed in an activity and perceives a close match between their personal skills and the challenges at hand (Wu and Liang, 2010). Individuals enter a flow state when they reach the highest level of concentration and motivation in intrinsically appealing activities (Nakamura and Csikszentmihalyi, 2014). A strong relationship exists between flow and motivation enhancement (Urhahne, 2008). Engaging in activities (e.g., exercise) inducing flow can bring individuals into an optimal state of participation or deep concentration. Individuals are presented to maintain their skills, experience smooth and coherent actions, and generate positive emotions in this state (Keller et al., 2025). Therefore, this may have important implications for individual wellbeing.

When individuals possess a high exercise motivation level, they perceive higher social support levels. A positive correlation exists between the two, which is beneficial for individuals’ mental health (Hui et al., 2023). Individuals with high exercise motivation levels exhibit greater interest, positive emotions, and enhanced attention during physical activity. Compared to those with low exercise motivation, they are more likely to perceive social support (Li et al., 2018).

Social support enhances an individual’s sense of belonging and self-worth, which fosters their interest and participation in physical activities and stimulates their intrinsic motivation (Li et al., 2023; Sulimani-Aidan and Melkman, 2022). Moreover, social support improves emotional regulation skills, self-efficacy, and an individual’s ability to persevere and cope with challenges, which promote exercise motivation and long-term participation (Xie, 2022).

Flow experience is context-dependent. There is relatively little empirical research on individuals’ flow experience under perceived social support. Currently, although researchers have explored the relationship between social support and flow experience, related research remains incomplete. Generally, flow experience is more common in competitive sports. Athletes with a higher perceived social support level exhibit a greater flow state (Stoll and Ufer, 2021), indicating a positive correlation between the two.

Social support is positively correlated with individuals’ positive subjective feelings, such as self-esteem and subjective wellbeing (Diener et al., 2009). If an individual’s social support network becomes stronger, more positive emotional experiences are provided, enabling them to better cope with various challenges (Cohen and Wills, 1985). This aligns with the essence of flow experience—the balance between challenges and skills.

Research on sports motivation and flow experience is relatively limited. Jackson’s work on elite athletes identified motivation as one of the psychological factors associated with the occurrence of flow, but did not establish motivation as the single most influential factor (Jackson, 1995). More recent flow research has also regarded high motivation, together with optimal challenge, as an important antecedent condition of flow (Brun et al., 2021). Csikszentmihalyi defined flow experience as a psychological state in which situational demands and an individual’s skills are at an equal level after studying flow experiences in various life domains such as work, leisure, and sports (Csikszentmihalyi and Csikszentmihalyi, 1988). Individuals become fully immersed in the activity in this state. Intrinsic motivation is defined as engaging in an activity driven by the enjoyment and satisfaction from the activity in Deci and Ryan’s research on the intrinsic and extrinsic motivation of human behavior (Deci and Ryan, 1985). Therefore, conceptually, the two are connected. Flow experience is associated with many positive outcomes, such as enhanced motivation and increased wellbeing (Keller and Bless, 2007; Schüler et al., 2012). This is also consistent with the findings of Peng (Peng et al., 2025). Based on self-determination theory, a positive correlation exists between sports motivation and flow experience.

According to social support theory, perceived social support is positively associated with individuals’ emotional experiences and psychological wellbeing (Zimet et al., 1988; Golden et al., 2009). From the perspective of self-determination theory, perceived social support may also be theoretically linked to enjoyment-based exercise motivation by supporting autonomy, competence, and relatedness (Hui et al., 2023). Enjoyment-based exercise motivation, in turn, is conceptually connected to flow experience because both involve interest, absorption, and satisfaction derived from the activity itself (Csikszentmihalyi and Csikszentmihalyi, 1988; Zawacki-Richter et al., 2019). Individuals reporting stronger flow experience also tend to report more positive emotions and higher wellbeing (Tse et al., 2020). Therefore, enjoyment-based exercise motivation and flow experience may serve as theoretically relevant mediating variables in the association between perceived social support and subjective wellbeing.

**Figure 1:**
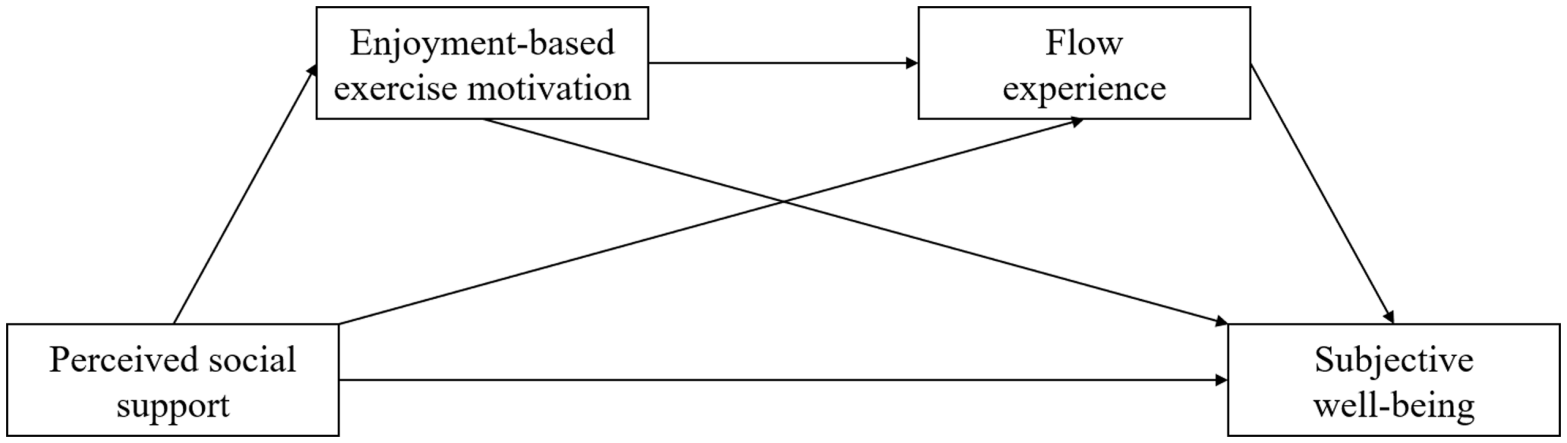
Hypothesized model.

The present study examined the relationships among perceived social support, enjoyment-based exercise motivation, flow experience, and subjective wellbeing in women who exercise regularly. Based on the aforementioned literature review, a structural model (Figure 1) was proposed. Five main hypotheses were developed based on this model.

*H1*: Perceived social support is positively associated with subjective well-being.

*H2*: Enjoyment-based exercise motivation and flow experience are positively associated with subjective well-being.

*H3*: Enjoyment-based exercise motivation statistically mediates the association between perceived social support and subjective well-being.

*H4*: Flow experience statistically mediates the association between perceived social support and subjective well-being.

*H5*: Enjoyment-based exercise motivation and flow experience show a chained indirect association between perceived social support and subjective well-being.

## 2 Materials and methods

### 2.1 Participants

A cross-sectional design was employed. A convenience sampling method was adopted, characterized by its simplicity and low cost. Questionnaires were administered to a total of 361 regularly exercising women in Shandong, Henan, Guangdong, and other provinces. The exclusion criteria for the questionnaires were as follows: (1) completion time of less than 180 s; (2) uniform responses across all items. A total of 23 participants were excluded from the data analysis (Gröschke et al., 2022), which resulted in a valid response rate of 94.01%.

In this study, “regularly exercising women” were operationally defined according to the World Health Organization physical activity recommendations for adults (Bull et al., 2020). Specifically, eligible participants were women who reported engaging in at least 150 min per week of moderate-intensity physical activity, or at least 75 min per week of vigorous-intensity physical activity, or an equivalent combination of moderate- and vigorous-intensity physical activity. This criterion was used during participant recruitment and screening.

Based on a Monte Carlo simulation, the work calculated the required sample size for the relevant model and back-calculated the effect size for the current chain mediation model. Minimum sample size *N* = 230 for the chain mediation model involving perceived social support and subjective wellbeing, with the power of 0.8. Required sample size *N* = 553 with the power of 1.0. The sample size of the current dataset was consistent with the projected sample size. Furthermore, the effect size of the reverse model indicated that our sample size (*n* = 361; *α* = 0.95) was adequate for indirect effect analysis (Schoemann et al., 2017). The questionnaire consisted of participant consent, demographic information, and research instruments. Among the 361 participants, 128 were aged 18–25 and 233 were aged 26–35, among the 361 questionnaires from regularly exercising female participants. Table 1 presents detailed information.

**Table 1:** Basic information of respondents (*n* = 361).

Characteristics		Frequency (*n*)	Effective percentage	Cumulative percentage
Age	18–25	128	35.5	35.5
	26–35	233	64.5	100.0
	(Total)	361	100.0	
Exercise duration (years)	1 and under	16	4.4	4.4
	1–3 years	248	68.7	73.1
	3 and above	97	26.9	100.0
	(Total)	361	100.0	
occupational category	Staff of government departments	88	24.4	24.4
	Enterprise employees	193	53.5	77.8
	Freelancers	80	22.2	100.0
	(Total)	361	100.0	

Data were collected with the cooperation and consent of the participants. The methods and precautions for completing the questionnaire were explained to regularly exercising women through both offline and online approaches. Before data collection, all participants were informed of their voluntary participation, anonymity, and the right to withdraw from the process at any point during the data collection.

The work strictly adhered to the principles outlined in the *Declaration of Helsinki* and obtained informed consent from all participants. Additionally, the approval was received from the Biomedical Ethics Committee of Qufu Normal University (Approval No. 2025128).

### 2.2 Methodology

Before data analysis, missing data and distribution were examined to assess the completeness of the entire dataset. Subsequently, validity and reliability were tested on all research data. Confirmatory factor analysis (CFA) was performed to evaluate the construct validity of all scales. This method was employed in place of exploratory factor analysis (EFA). The factor structures of all instruments have been well-established and extensively reported in prior research, which eliminated the need for further exploration of latent factors. The structural validity was evaluated based on model fit indices, including the *χ*^2^/df statistic, comparative fit index (CFI), adjusted goodness of fit index (AGFI), Tucker-Lewis index (TLI), root mean square error of approximation (RMSEA), and standardized root mean square residual (SRMR). All analyses were performed using structural equation modeling (SEM) within the statistical package for the social sciences (SPSS) and the analysis of moment structures (AMOS).

#### 2.2.1 Independent variable

Perceived social support is measured using the Chinese version of the Perceived Social Support Scale, translated and revised by Qianjin J. (Jiang, 2001). The revised scale contains 12 items and three dimensions (e.g., family support, friend support, and other support). Higher scores indicate a higher overall level of perceived social support. Cronbach’s *α* = 0.726. The model fit indices are as follows after revision: *χ*^2^/df = 3.462, GFI = 0.945, AGFI = 0.905, CFI = 0.840, TLI = 0.775, RMSEA = 0.083, and SRMR = 0.079.

#### 2.2.2 Dependent variable

Subjective wellbeing is measured using the Satisfaction with Life Scale (SWLS) developed by Diener et al. (1985), which assesses participants’ perceived life satisfaction. Serving as a key indicator of subjective wellbeing, life satisfaction is a more stable and affirmative measure independent of positive and negative affect (Pavot and Diener, 2009). The SWLS primarily captures life satisfaction, the cognitive component of subjective wellbeing, whereas affective components such as positive and negative affect were not assessed. It reflects individuals’ overall evaluation and attitude toward their life quality, aligning with the long-term psychological benefits associated with regular exercise. Using a 7-point Likert scale, the scale consists of 5 items, such as “In most ways, my life is close to my ideal.” Higher scores indicate greater life satisfaction. Cronbach’s *α* = 0.801. After revision, *χ*^2^/df = 2.779, GFI = 0.985, AGFI = 0.954, CFI = 0.981, TLI = 0.962, RMSEA = 0.070, and SRMR = 0.033.

The SWLS primarily captures life satisfaction, the cognitive component of subjective wellbeing, whereas affective components such as positive and negative affect were not assessed.

#### 2.2.3 Mediating variable (i)

The Chinese version of the Motivation for Physical Activity Measure (MPAM), revised by Chen et al. (2013), is used. This scale consists of 15 items across 5 dimensions (i.e., health motivation, competence motivation, enjoyment motivation, appearance motivation, and social motivation). Higher scores indicate a higher level of individual exercise motivation. Cronbach’s *α* = 0.702 in the questionnaire. After adaptation, *χ*^2^/df = 4.967, GFI = 0.936, AGFI = 0.880, CFI = 0.841, TLI = 0.762, RMSEA = 0.105, and SRMR = 0.080.

In the present study, enjoyment-based exercise motivation was specified as the focal motivational construct because it represents the intrinsic motivational component most closely aligned with self-determination theory and flow experience. Specifically, enjoyment-based exercise motivation reflects participation in exercise for pleasure, interest, and satisfaction derived from the activity itself. Therefore, this subscale was used as the motivational mediator in the final structural model.

For the enjoyment-based exercise motivation subscale, the standardized factor loadings ranged from 0.57 to 0.72. The subscale showed acceptable measurement evidence for a short three-item construct, with Cronbach’s *α* = 0.661 and CR = 0.665. Although the AVE was 0.401, slightly below the conventional 0.50 criterion, recent methodological literature recommends that reliability and validity should be evaluated using multiple sources of evidence rather than relying solely on Cronbach’s alpha or a single cutoff value (Boateng et al., 2018). Therefore, considering the theoretical relevance of enjoyment-based motivation within self-determination theory, the moderate standardized factor loadings, and the short length of the subscale, this construct was used as the motivational construct in the final model.

#### 2.2.4 Mediating variable (ii)

The Chinese version of the State Flow Experience Scale (FSS-2), revised by Liu (2012), is adopted. The scale consists of 33 items across nine dimensions (i.e., balance, merging of action and awareness, clear goals, unambiguous feedback, concentration, sense of control, loss of self-consciousness, transformation of time, and autotelic experience). Participants recall an experienced activity and rate it based on their personal experience. Higher scores indicate a higher level of state flow experience. Cronbach’s *α* = 0.809. For the scale, *χ*^2^/df = 1.313, GFI = 0.919, AGFI = 0.904, CFI = 0.939, TLI = 0.932, RMSEA = 0.030, and SRMR = 0.050.

### 2.3 Data analysis

SPSS 26.0 was used for data cleaning, common method bias testing, descriptive statistics, and correlation analysis. Based on the above correlation analyses and the proposed hypotheses, a chain mediation model was established to examine the relationship between perceived social support and subjective wellbeing among women who exercised regularly. AMOS 29.0 was employed to test the chain mediation pathway involving enjoyment-based exercise motivation and flow experience. The work employed maximum likelihood (ML) estimation and conducted bootstrap resampling with 5,000 iterations for model estimation, with 95% confidence intervals (CIs) calculated accordingly.

## 3 Results

### 3.1 Common method bias test

The Harman single-factor test was applied to all items of the four key variables in the work context to examine common method bias (Chang et al., 2010). The first factor accounted for 17.11% of the variance, which was below the 40% threshold. Thus, common method bias did not have a significant impact.

### 3.2 Descriptive statistics

Table 2 presents descriptive statistics (i.e., means and standard deviations), bivariate correlations, and internal consistency reliability estimates for the main variables. All variables demonstrate acceptable and good internal consistency reliability. Correlation analyses reveal that perceived social support is positively correlated with enjoyment-based exercise motivation (*r* = 0.188 and *p* < 0.001), flow experience (*r* = 0.179 and *p* < 0.001), and subjective wellbeing (*r* = 0.440 and *p* < 0.001). Furthermore, both enjoyment-based exercise motivation (*r* = 0.444 and *p* < 0.001) and flow experience (*r* = 0.464 and *p* < 0.001) are positively correlated with subjective wellbeing. These results provide preliminary evidence for further testing the hypothesized chain mediation model.

**Table 2:** Correlation analysis.

Variables	Descriptive statistics			Correlation			
	*M*	SD	*α*	1	2	3	4
1. Perceived social support	3.97	0.34	0.726	1			
2. EBEM	4.97	0.40	0.661	0.1872^***^	1		
3. Flow experience	4.25	0.24	0.809	0.179^***^	0.1867^***^	1	
4. Subjective wellbeing	5.06	0.51	0.801	0.440^***^	0.444^***^	0.464^***^	1

EBEM, enjoyment-based exercise motivation. EBEM is a short three-item subscale; its reliability and validity evidence is reported in the measures section.

### 3.3 Statistical mediation analysis

**Figure 2:**
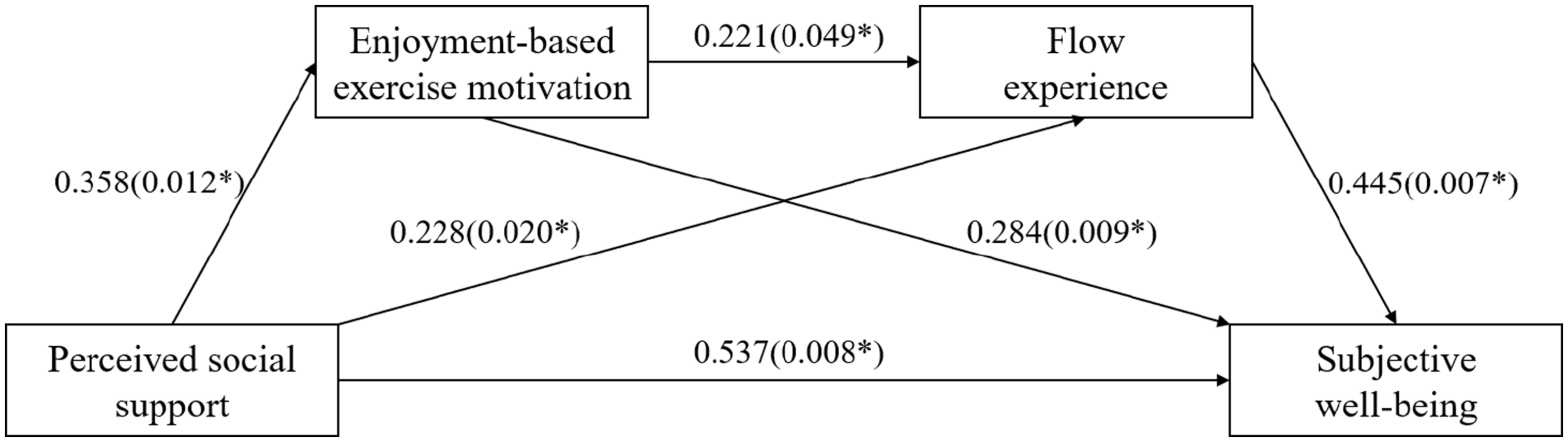
Structural mediation model of the associations among perceived social support, enjoyment-based exercise motivation, flow experience, and subjective wellbeing.

Statistical mediation was examined using path analysis in AMOS version 29.0. The Bootstrap method was employed to test the mediating effects, with perceived social support specified as the focal explanatory variable, enjoyment-based exercise motivation and flow experience as statistical mediators, and subjective wellbeing as the outcome variable. Specifically, bias-corrected bootstrap CIs (5,000 iterations) were constructed in AMOS to examine the proposed indirect association hypotheses. A statistically significant indirect association was indicated if the 95% CI did not include zero. Figure 2 and Table 3 present the results.

**Table 3:** Statistical mediation analysis.

Path/Effect type	*β*	Sig	LLCI	ULCI
Direct effects paths				
PSS → EBEM	0.358	0.012	0.185	0.521
PSS → FE	0.228	0.020	0.048	0.466
PSS → SWB	0.537	0.008	0.420	0.672
EBEM → FE	0.221	0.049	0.005	0.437
EBEM → SWB	0.284	0.009	0.123	0.411
FE → SWB	0.445	0.007	0.303	0.592
Effect type	Estimate			
Total	0.775	0.016	0.663	0.869
Direct	0.537	0.008	0.420	0.672
Indirect	0.238	0.011	0.144	0.358
Ind1 (PSS → EBEM → SWB)	0.102	0.005	0.044	0.179
Ind2 (PSS → FE → SWB)	0.101	0.013	0.028	0.198
Ind3 (PSS → EBEM → FE → SWB)	0.035	0.022	0.006	0.108

PSS, perceived social support; SWB, subjective wellbeing; EBEM, enjoyment-based exercise motivation; FE, flow experience.

The structural equation model was established to examine the direct and indirect associations between perceived social support and subjective wellbeing among regularly exercising women. The model showed acceptable fit: *χ*^2^/df = 1.208, GFI = 0.907, AGFI = 0.893, CFI = 0.951, TLI = 0.947, and RMSEA = 0.024. Perceived social support was positively associated with enjoyment-based exercise motivation, flow experience, and subjective wellbeing. Enjoyment-based exercise motivation was positively associated with flow experience and subjective wellbeing, and flow experience was positively associated with subjective wellbeing.

The total association between perceived social support and subjective wellbeing was 0.775, with a direct association of 0.537 and an indirect association of 0.238. The bootstrap results indicated three statistically significant indirect pathways: perceived social support → enjoyment-based exercise motivation → subjective wellbeing; perceived social support → flow experience → subjective wellbeing; and perceived social support → enjoyment-based exercise motivation → flow experience → subjective wellbeing.

Specifically, perceived social support was positively associated with enjoyment-based exercise motivation (*β* = 0.358 and *p* < 0.05), flow experience (*β* = 0.228 and *p* < 0.05), and subjective wellbeing (*β* = 0.537 and *p* < 0.05), which supports hypothesis 1. Both enjoyment-based exercise motivation (*β* = 0.284, *p* < 0.01) and flow experience (*β* = 0.445, *p* < 0.01) were positively associated with subjective wellbeing.

Perceived social support showed indirect associations with subjective wellbeing among regularly exercising women through two pathways: enjoyment-based exercise motivation (indirect effect ind1 = 0.102; 95% CI [0.044, 0.179]) and flow experience (indirect effect ind2 = 0.101; 95% CI [0.028, 0.198]). This supported hypothesis 3 and 4. Perceived social support also showed a chained indirect association with subjective wellbeing (indirect effect ind3 = 0.035; 95% CI [0.006, 0.108]). Enjoyment-based exercise motivation and flow experience functioned as statistical mediators in the model.

## 4 Discussion

Based on self-determination theory, a statistical chain mediation model was constructed to examine the potential psychological pathways among perceived social support, enjoyment-based exercise motivation, flow experience, and subjective wellbeing. The model indicated associations between perceived social support and the subjective wellbeing of regularly exercising women, as well as the mediating roles of enjoyment-based exercise motivation and flow experience. This initiative served as a proactive exploration of future support strategies aimed at addressing potential risks and supporting the psychosocial and physiological health of this demographic.

The theoretical contribution of this study lies in refining the explanation of how perceived social support is associated with subjective wellbeing among women who exercise regularly. Rather than proposing entirely new constructs, the study integrates perceived social support, enjoyment-based exercise motivation, and flow experience into a theoretically grounded model. In particular, enjoyment-based exercise motivation reflects the intrinsic motivational component emphasized in self-determination theory and helps explain why socially supportive environments may be associated with more positive exercise experiences. The inclusion of flow experience further connects motivational quality with subjective wellbeing, thereby extending prior research that has examined these constructs separately. At the practical level, the importance of perceived social support is highlighted. The potential indirect associations between perceived social support and subjective wellbeing were examined through enjoyment-based exercise motivation and flow experience. Furthermore, new insights are provided for enhancing wellbeing.

### 4.1 Relationship between perceived social support and subjective wellbeing in women who exercise regularly

The wellbeing of women is attracting increasing attention with the continuous development of society. As more and more women engage in exercise, their subjective wellbeing has garnered greater interest from researchers. Subjective wellbeing reflects an individual’s satisfaction with life, serving as a protective factor for their mental health. Correlation analyses and SEM indicate that subjective wellbeing was significantly and positively associated with the level of perceived social support among women who exercise regularly. This finding is consistent with those of other researchers, demonstrating that social support has been positively associated with life satisfaction in these women. Supportive relationships with family and friends are effective ways to foster their subjective wellbeing (Harknett, 2006; Ryan et al., 2009). Individuals with a high level of perceived social support can experience positive benefits (Lin, 2001).

One of the seven determinants/correlates of subjective wellbeing is social support, with others including basic demographic characteristics, finances, health, personality, culture, geographical location, and infrastructure (Das et al., 2020). The three variables of perceived social support (e.g., family support, peer support, and other support) are validated to be consistent with the findings of previous publications in the work. The quality of interpersonal relationships is positively associated with subjective wellbeing (Pinquart and Sörensen, 2000; Sandstrom and Dunn, 2014). Perceived social support has been associated with subjective wellbeing, lower perceived stress, and life satisfaction.

### 4.2 Mediating roles of enjoyment-based exercise motivation and flow experience

Perceived social support showed an indirect association with subjective wellbeing of women who exercise regularly through enjoyment-based exercise motivation and flow experience. Both factors serve as independent and sequential mediators. The chain-mediating effect involving enjoyment-based exercise motivation and flow experience is a key finding of the work, which has not been confirmed in previous research. These findings provide a new perspective on the potential pathways linking perceived social support and subjective wellbeing.

Self-determination theory categorizes intrinsic motivation into three sub-dimensions: enjoyment/pleasure, achievement/challenge, and learning/exploration. Enjoyment/pleasure serves as a foundational component of intrinsic motivation, most conducive to sustained behavioral adherence and positive engagement (Vallerand, 2000). Perceived social support was indirectly associated with subjective wellbeing through enjoyment-based exercise motivation. For women who exercise regularly, compared to extrinsic motivations such as health or appearance, enjoyment-based exercise motivation may be related to more active engagement in exercise. This engagement may also be associated with a greater likelihood of experiencing flow and with higher subjective wellbeing.

This aspect constitutes a gap in previous research. Prior studies have examined exercise motivation as a holistic construct when exploring its mediating effects. In contrast, the work focuses on the sub-dimension of enjoyment-based exercise motivation. For different populations, not all motivational types function as mediators. The finding enriches the application of self-determination theory in physical exercise, providing a more specific reference point for future interventions or support strategies related to women’s exercise motivation.

Perceived social support may help satisfy the emotional and relational needs of female exercisers. Enjoyment-based exercise motivation may emerge as an intrinsic motivational tendency associated with the fulfillment of these needs. Such motivation may help individuals concentrate on the exercise process and become more likely to experience a flow state when there is an optimal challenge-skill match. The positive emotions associated with this process may be further linked to subjective wellbeing.

Our findings may have practical implications for supporting life satisfaction and wellbeing of women who exercise regularly. Perceived social support was positively associated with the subjective wellbeing of women who exercise regularly; thus, a diversified social support system tailored for female exercisers may be considered. Family members, friends, and other close relationships could be encouraged to provide emotional support, such as actively joining them in exercise. Establishing women-only fitness communities or social settings may help strengthen a sense of belonging through peer support. In this way, perceived social support may be transformed from sporadic emotional encouragement into a systematic support network, which may provide a supportive social context for wellbeing.

In light of the mediating roles of enjoyment-based exercise motivation and flow experience, future strategies may place greater emphasis on the element of enjoyment during exercise. Reframing the logic for motivating women to exercise involves adaptively reducing outcome-driven messaging centered solely on weight loss or body shaping and shifting the focus toward the inherent enjoyment of the exercise process. This approach may help women experience a sense of control, enjoyment, and accomplishment during exercise and may support a shift from passively persisting in workouts to actively embracing the joy of movement. Enjoyment-based exercise motivation may represent a potential psychological pathway linking exercise participation with wellbeing.

## 5 Conclusion

The following conclusions can be drawn. First, the work reveals the positive correlations among perceived social support, enjoyment-based exercise motivation, flow experience, and subjective wellbeing. Second, compared to previous research, perceived social support was directly associated with subjective wellbeing in female exercisers. Besides, subjective wellbeing is indirectly related to the independent and sequential indirect associations of enjoyment-based exercise motivation and flow experience. This enriches the theoretical framework linking perceived social support and subjective wellbeing. In particular, examining the statistical mediating roles of enjoyment-based exercise motivation and flow experience contributes to our understanding of how perceived social support may be linked to women’s intrinsic emotional needs. A novel perspective is provided for future research in this field.

Four main limitations should be acknowledged. First, the relationships observed are correlational rather than causal because of the cross-sectional design. Future studies should use longitudinal, repeated-measurement, or experimental designs to better establish temporal order and causality. Second, the findings may be influenced by common method bias or social desirability bias because all data were collected through self-reported questionnaires. Third, enjoyment-based exercise motivation was measured using a short three-item subscale with relatively modest psychometric indicators, as reported above. Although this does not invalidate the findings, future studies should examine this motivational dimension using more comprehensive measures with stronger psychometric properties. Fourth, the sample consisted only of women who exercise regularly, and detailed exercise characteristics such as weekly frequency, session duration, and exercise modality were not collected. Future research should include more diverse groups and more detailed exercise-related information to improve the generalizability and contextual interpretation of the findings.

## Data Availability

The original contributions presented in the study are included in the article/supplementary material, further inquiries can be directed to the corresponding author.
